# The gordian knot of overlapping symptoms between dissociative identity disorder and borderline personality disorder, the need for a clear cut: A case report

**DOI:** 10.1192/j.eurpsy.2021.2039

**Published:** 2021-08-13

**Authors:** I. Romanos, M. Preve, R. Traber

**Affiliations:** 1 Outpatient Psychiatric Service, Sociopsychiatric Organization, Mendrisio, Switzerland; 2 Inpatient And Outpatient Psychiatric Service, Sociopsychiatric Organization, Mendrisio, Switzerland

**Keywords:** tertiary structural dissociation, Borderline personality disorder, Dissociative Identity Disorder, overlapping symptoms

## Abstract

**Introduction:**

One of the central debates in the psychiatric community is the difficulty in distinguishing Dissociative Identity Disorder (DID) from Borderline Personality Disorder (BPD). The fact that core symptoms of these pathologies such as emotional dysregulation, alterations in sense of Self, amnesia, depersonalization, self harm, hearing voices, difficulties in maintaining relationships, are symptoms that feature in both disorders can lead physicians to a misdiagnosis, thus depriving patients with DID of adequate treatment.

**Objectives:**

To report a complex clinical case of a DID patient initially misdiagnosed as BPD.

**Methods:**

Clinical case report.

**Results:**

A 45-year-old Caucasian woman with a history of childhood intrafamilial sexual abuse and domestic violence, substance use disorder, autolesionistic and suicidal behaviour with an active diagnosis of BPD presented to our ambulatory mental health care service. A more thorough examination revealed a history of emotional and affect dysregulation, depersonalization, amnesia, intrusive traumatic memories and nightmares with affective, cognitive, and sensorimotor aspects, persistent negative Self-perception. Auditory verbal hallucinations were also present described as inner space with commentary and derogatory nature with one of them being a child voice. The diagnosis of tertiary structural dissociation and DID was finally made when three Apparently Normal Personalities emerged with several Emotional Personalities, authorising for cautious partial pharmacological washout and initiation of three phase-orientated treatment approach.

**Conclusions:**

DID is more common than is assumed and the overlap of core symptoms with other disorders can lead to a misdiagnosis. A careful clinical interview and evaluation of symptoms is mandatory to a correct DID diagnosis with a consequent appropriate therapy.

**Disclosure:**

No significant relationships.

